# Baseline and Estimated Trends of Sodium Availability and Food Sources in the Costa Rican Population during 2004–2005 and 2012–2013

**DOI:** 10.3390/nu9091020

**Published:** 2017-09-15

**Authors:** Adriana Blanco-Metzler, Rafael Moreira Claro, Katrina Heredia-Blonval, Ivannia Caravaca Rodríguez, María de los A. Montero-Campos, Branka Legetic, Mary R. L’Abbe

**Affiliations:** 1 Costa Rican Institute of Research and Training in Nutrition and Health, Tres Rios 4-2250, Costa Rica; mmontero@inciensa.sa.cr; 2 Nutrition Department (NUT), Federal University of Minas Gerais (UFMG), Belo Horizonte MG 30.130-100, Brazil; Rafael.claro@gmail.com; 3 Independent Nutritionist, San José 10203-1000, Costa Rica; katrihe@gmail.com; 4 Ministry of Health, San José 10123-1000, Costa Rica; ivannia.caravaca@misalud.go.cr; 5 Independent Consultant, Novi Sad 21000, Serbia; legeticb@gmail.com; 6 Department of Nutritional Sciences, University of Toronto, Toronto, ON M5S 3E2, Canada

**Keywords:** salt, sodium, population intervention, policy, food consumption, socioeconomic factors, Costa Rica, Latin America

## Abstract

In 2012, Costa Rica launched a program to reduce salt and sodium consumption to prevent cardiovascular disease and associated risk factors, but little was known about the level of sodium consumption or its sources. Our aim was to estimate the magnitude and time trends of sodium consumption (based on food and beverage acquisitions) in Costa Rica. Data from the National Household Income and Expenditure Surveys carried out in 2004–2005 (*n* = 4231) and 2012–2013 (*n* = 5705) were used. Records of food purchases for household consumption were converted into sodium and energy using food composition tables. Mean sodium availability (per person/per day and adjusted for a 2000-kcal energy intake) and the contribution of food groups to this availability were estimated for each year. Sodium availability increased in the period from 3.9 to 4.6 g/person/day (*p* < 0.001). The income level was inversely related to sodium availability. The main sources of sodium in the diet were domestic salt (60%) in addition to processed foods and condiments (with added sodium) (27.4%). Dietary sources of sodium varied within surveys (*p* < 0.05). Sodium available for consumption in Costa Rican households largely exceeds the World Health Organization-recommended intake levels (<2 g sodium/person/day). These results are essential for the design and implementation of effective policies and interventions.

## 1. Introduction

Recent data on sodium intake show that populations around the world are consuming an amount of sodium that is excessive to what is physiologically necessary. In many cases, intake also exceeds the current World Health Organization (WHO) recommendations on sodium consumption for adults, which is 2 g sodium/day (equivalent to 5 g salt/day) [1,2]. A direct relationship between excessive sodium intake and the development of hypertension exists, which is responsible for 30% of the hypertension burden worldwide [3].

Globally, high blood pressure (hypertension) is the main risk factor for and the leading cause of death as well as being the second risk for disability related to cardiovascular disease (CVD) [4,5]. An estimated 17.9 million people died from CVD in 2015, representing 31% of all deaths. Over three quarters of CVD deaths take place in low- and middle-income countries [6]. 

In Costa Rica, a middle-income country, CVDs represent the leading cause of death since 1970 [7,8]. Two surveys of cardiovascular risk factors based on the Stepwise methodology of the Pan American Health Organization/World Health Organization (PAHO/WHO) have been recently carried out in the country [7,9], showing similar results for hypertension. The national prevalence of hypertension in adults over 19 years of age has remained constant at 37.8% in 2010 and 36.2% in 2014 (no significant difference) [9].

Reducing sodium intake is recognized as the most cost-effective intervention in preventing hypertension, CVD, and several other related conditions [10,11]. This is the main reason for the PAHO/WHO launching the “Cardiovascular Disease Prevention Initiative by Reducing Salt Consumption in the Americas” in 2009. This policy statement established a gradual reduction of sodium intake to reach a target of 2 g per person per day by 2020 (or 5 g of salt/person/day) [10].

Sodium intake can be estimated by a wide range of methods. According to PAHO, the Household Budget Survey (HBS) methodology represents a viable option to estimate sodium consumption by household members in countries with limited economic resources [10]. Brazil [12,13], Poland [14], and Slovenia [15] have used this method to measure salt intakes and identify the main dietary sources. 

The objective of this study was to estimate the magnitude, distribution, and time trends in the availability of sodium in households of Costa Rica as well as the main food sources by analyzing the HBS conducted in Costa Rica during the years of 2004–2005 and 2012–2013. This scientific evidence is essential for the development of the sodium reduction national program and to achieve the salt goal of the WHO Global Action Plan for the Prevention and Control of Non-Communicable Diseases 2013–2020 [16].

## 2. Materials and Methods

The design of the study is ecological. The data analyzed were collected during the National Household Income and Expenditure Surveys (ENIGH) performed during 2004–2005 (ENIGH 2004–2005) and 2012–2013 (ENIGH 2012–2013). ENIGH is the specific name given to the HBS in Costa Rica. These surveys were carried out by the National Institute of Statistics and Censuses of Costa Rica (INEC). These surveys provide information on the composition of the budget in the country's households through knowledge of their income and expenditures on goods and services.

A probabilistic sampling design was employed, resulting in data that is representative of all households in Costa Rica and in the two zones of the country (urban and rural households). The sampling was based on a complex strategy that applies the previous definition of the socioeconomic strata and integrates the 348 (ENIGH 2004–2005) and 468 (ENIGH 2012–2013) sectors in the same territorial domain (zone and region) in strata that are economically homogeneous. Following this, sectors were selected in each stratum and households were selected within each sector. Finally, in order to standardize the data collection in the four quarters of the year, interviews were conducted in each sector throughout the 12 months of the study. A detailed description of the ENIGH sampling strategy is available in previous studies [17,18].

The records of all food and beverage purchases by the household for a period of seven consecutive days were analyzed (in ENIGH, the weekly records are converted to a month for the end registers). More details about ENIGH methodology can be found in previous studies [17,18]. A total of 96,336 purchases (by 4231 households) were recorded in ENIGH 2004–2005 and 186,308 (by 5705 households) in ENIGH 2012–2013. Information regarding the acquisition of foods outside the home was not available in ENIGH 2004–2005 and in 2012–2013, although the acquisition of snacks outside the home were registered. Foods outside the home corresponds to food items purchased and consumed outside the home, with snacks corresponding to one type of these food items. The criterion adopted for the recording of expenditure in ENIGH is “acquired”, as the information available for each household is a proxy measure and not “actual intake”.

The income per household used in both HBSs is the total net income of the household and includes the income that the household members receive in an average month for the last 12 months. This includes income from wages; self-employment with deductions from law; financial assets and rental of properties; transfers in cash or in kind; imputed rent (non-monetary income of the value of the imputed rental of own housing); as well as financial or capital transactions [17,18].

The methodology developed by Monteiro [12,13] consists of converting the food and beverage acquisition records of the family budget survey into nutrients by means of food composition tables. A national food composition table was constructed by collecting the sodium and energy contents of approximately 980 foods and typical recipes. Due to the lack of sodium content data in the national food composition tables, the United States Department of Agriculture's (USDA) nutrient database [19] was used as the main data source. In the case of fortified foods and native foods, the tables of food composition published by the Costa Rican Institute for Research and Teaching in Nutrition and Health [20,21] were used. To determine the sodium content in typical Costa Rican recipes, the database of the Nutrition School of the University of Costa Rica ValorNut [22] was used. The nutritional content of food recipes or preparations was calculated based on the methodology established by the Food and Agriculture Organization of the United Nations [23]. A comprehensive review of the built table was performed and food was classified into the five food groups established by the research team.

The availability of sodium/person/day and the contribution for each food group was calculated. The results of ENIGH 2012–2013 were compared with those of ENIGH 2004–2005 before trends were established.

Items were grouped based on the categories established by Monteiro [12,24], which consisted of separating common salt from salt-based condiments. The final classification was defined by five categories: common salt; condiments with added sodium; processed foods without added sodium; prepared dishes; “natura” (foods in their natural state, without any processing), and processed foods with added sodium (excluding condiments).

Food and beverage acquisition records (containing the amount acquired in kilograms or liters) from ENIGH 2004–2005 and 2012–2013 were initially linked to sodium and energy content. The net quantity of each product was estimated by removing the non-edible fraction from the total acquired quantity (gross quantity) and used in the determination of the sodium amount of each acquisition. Following this, the availability of sodium was estimated (per capita/day, by dividing the total acquired quantity by the number of people in the household and by 30.33 (average number of days per month) for the entire set of foods and beverages, according to food groups. This was estimated for the total population and according to zone (rural and urban) and income level (using *t*-test). 

As this survey is not designed for nutritional purposes, and in order to mitigate errors in the analysis and allow for comparisons between surveys, the results were standardized to 2000 kcal, which is the average energy consumption of a healthy adult [25]. 

Consumption trends were identified and compared between both surveys. The surveys are methodologically comparable except that in ENIGH 2004–2005, the sample does not allow stratification at the regional level (due to inconsistencies in some regional information, INEC decided not to recommend this stratification). This comparison was conducted for the entire population of each survey and according to income levels. For this, the per capita income was first estimated in each household (by dividing total income by the number of individuals in each household using information available at ENIGH) and used to divide the population into five income levels (based on the quintiles of per capita income distribution). Student’s *t*-tests were used to compare each income level between the surveys, while a regression model (Generalized Linear Model was used to investigate trends between the income levels within each survey.

Analyzes were performed with the statistical program SPSS version 20 (IBM Corp, New York, NY, USA). In each unit of analysis, the expansion factor was considered. This factor is obtained as the inverse of the probability of selecting each house at the time of selecting the sample. We tested the hypothesis at the significance level of 5% by the Student’s *t*-test [26].

## 3. Results

Household energy and sodium availability in Costa Rica, unadjusted and adjusted to 2000 kcal, is shown in Table 1 according to the zone and year of survey. The amount of sodium available for consumption in 2004–2005 and 2012–2013 was 3.9 and 4.6 g/person/day, respectively. A statistically significant increasing trend was found (*p* < 0.0001). In both surveys, a significant difference (*p* < 0.0001) was found between the availability of sodium in rural compared to urban areas. In all areas, the available sodium was always greater than 3.6 g/person/day. Adjusting the sodium availability to a 2000-kcal diet did not substantially alter the scenario. The available sodium analysis of ENIGH 2012–2013 showed no significant differences between regions (*p* > 0.05).

**Figure 1:**
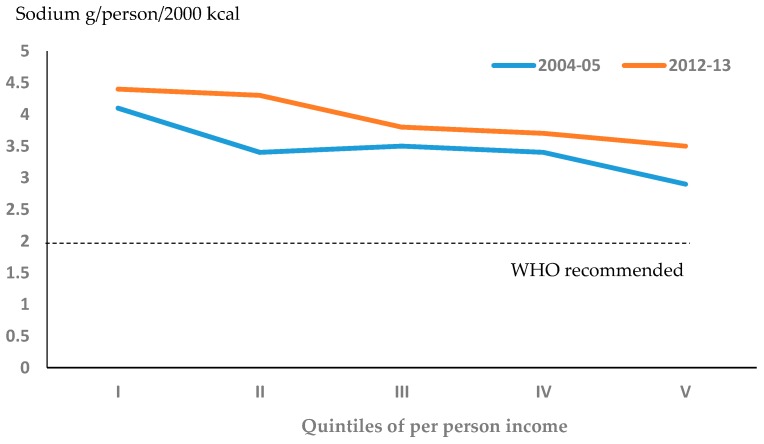
Trends in household availability of sodium * according to increasing quintiles of per person family income in Costa Rica with a comparison of the data from 2004–2005 and 2012–2013. * Adjusted to a 2000-kcal diet. For more information, see Section 2.

The energy and sodium available in households according to income levels (quintiles) is shown in Table 2. In both surveys, all income quintiles exceeded the maximum recommended intake of sodium (2 g/person/day) with a minimum of 3.8 g/person/day in 2012–2013, which had no linear relation with income level in the unadjusted data. However, the analysis of energy values showed that the sodium intake had an inversely proportional relationship with the income (Figure 1). On the other hand, when comparing sodium availability (g/person/day) surveys conducted during 2004–2005 and 2012–2013, we found differences in sodium available between quintiles II, IV, and V (*p* < 0.05), with none found between quintiles I and III (*p* > 0.05).

Table 3 shows the sources of sodium in the diet of the population of Costa Rica by household per person income. Common salt (table or kitchen salt) was the main source, with an estimated intake of 2.4 and 2.8 g/person/day in the ENIGH 2004–2005 and 2012–2013, respectively. In both surveys, common salt contributes 60% of total sodium available in the households, followed by processed foods and condiments. The dietary source that contributed least to dietary sodium was natural and processed foods with no added sodium, which accounted for only 5% of sodium availability. Statistical differences (*p* < 0.05) were found between the food sources of sodium in both surveys. A significant trend over time (*p* < 0.05) was found due to an increase in the sodium intake per person from processed foods and condiments with sodium added. In comparison, in ready-to-eat meals and natural foods, the sodium availability tended to decrease or remain constant between surveys. Socioeconomic income was inversely associated with common salt availability, as well as being directly associated with a greater acquisition of processed foods, natural foods, and prepared dishes (*p* < 0.05). No association was found between socioeconomic income and acquisition of condiments. 

## 4. Discussion

The household availability of sodium in Costa Rica is within the internationally reported range (3.6 to 4.8 g/person/day) [10], but exceeds the WHO maximum intake recommendation [2]. The higher amount of sodium available in the rural areas can be explained by the greater availability of energy and, therefore, of foods that provide sodium. Other authors have reported that the consumption of salt is directly proportional to energy [12]. 

We found that there was a trend of increased sodium acquisition in households over time, with a 15% increase in per person household acquisition in less than a decade. This result is extremely relevant for public health, as it indicates the urgent need for effective actions capable of stopping this expansion and methods aiming to reduce sodium consumption in the country. The results of the present study also indicate that Costa Rica, up to 2013, was failing to meet the goal set out in the “National Strategy for Comprehensive Management of Chronic Non-Communicable Diseases and Obesity 2014–2021”, which was an average relative reduction of “15% in daily salt/sodium intake” [27]. This goal was based on the sodium available in the homes estimated in the ENIGH 2004–2005 [18] and, until the publication of this study, no results from ENIGH 2012–2013 were available.

This study is the first one that estimates sodium availability in Costa Rican households at a national level. Previously, the consumption of sodium in Costa Rica has only been estimated in the metropolitan areas. Using the method of seven-day food diaries, the mean consumption of sodium was estimated as 3.6 g/person/day in the metropolitan city of Cartago [28]. Using the technique of 24-h urine in a sample of 30 adult men with hypertension in San Jose, the capital of the country, sodium intake was found to be 3.8 g/person/day [29]. These studies estimated 0.8 and 1.0 g/person/day less sodium than what we estimated in the HBS 2012–2013. Differences may be due to the methodology used, the size of the populations, and their representativeness, as more sodium is consumed in the rural population [12,13]. The amount of domestic salt estimated in the present study is within the range reported in previous studies conducted in the country [28,29,30]. 

Despite differences in the methodology used to estimate sodium consumption [28,29], all studies show that the consumption of sodium and of common salt (kitchen or table) in Costa Rica considerably exceeds the international recommendation [2].

An inverse relationship was found between sodium availability in households (energy-adjusted) and income, which is similar to that reported in studies conducted in developing [12,13] and developed countries [31]. Although a poorer diet quality among low-income families may be one of the reasons for high sodium intake [31], this result might also relate to the main source of sodium consumption in Costa Rica, namely common salt (table or kitchen).

Regarding the main sources of dietary sodium, income level is known to play a major influence. In developed countries, the main sources of sodium are processed foods and meals outside the home. In addition, a smaller contribution of common salt and condiments has been reported [32], since cooking in the home is less common in developed countries [33]. In Costa Rica and Brazil, food is still prepared in home, with common salt and condiments representing the main sources of sodium in the diet [13]. However, in both countries, there is has been increasing availability of ultra-processed foods seen in recent years [24,34].

Contrary to expectations, the contribution of prepared dishes on the household availability of sodium decreased between surveys (from 9.8% to 7.2%). These differences are due to changes in food collection and classification between two surveys in a way that resulted in the comparison for this food group not being recommended. Natural and processed foods without added sodium represented the groups that provide less sodium to the diet. This behavior is similar in both surveys and shows that, unfortunately, the acquisition of natural foods did not increase in this period. The reduced household availability of natural foods is directly associated with a better economic income in Costa Rica. 

Condiments, mainly consommés and cubes, was the food group whose sodium contribution to diet increased most over the course of nine years, with their availability in households having almost doubled. In the late 1990s, the Costa Rican Ministry of Health found an important substitution of domestic salt for condiments in household daily food preparations. For this reason, as a part of the salt fortification policy, the government established that salt used in the manufacture of consommés and cubes must contain iodine and fluorine at the levels stipulated for domestic salt since 2001 [35]. This policy was designed to prevent an iodine deficiency in the population and it may have contributed to the increase in the acquisition of commercial condiments high in salt. However, as we demonstrate in this study, there has been an increase not only in the intake of condiments but also in domestic salt. 

The results of the present study serve as an important driver for the inclusion of condiments in the preparation of the PAHO regional targets on sodium reduction [36]. They also have been used in the establishment of the national goal of salt reduction; the preparation of the action plan of the national strategy for the management of chronic non-communicable diseases in Costa Rica [27]; and the establishment of national sodium reduction targets in key processed foods.

The HBS method has its limitations, because the survey was designed for economic reasons and not for nutritional purposes. Due to this ecological type of study, there are several main limitations. Firstly, our method overestimates the consumption of sodium because it is assumed that every food and beverage item purchased is for human consumption. Secondly, it is impossible to estimate the consumption of sodium away from home, since only food and beverages acquired for household consumption are recorded with enough information. Thirdly, the use of food composition tables does not always precisely estimate the sodium content of the foods consumed by participants. Furthermore, most of the data for the sodium content of foods was determined from the food composition table of the USDA, with a lack of updated local food data. There needs to be further confirmation with more precise studies, especially considering that a homogenous distribution is assumed in this present study. However, the advantage of this ecological method is economical, because the data already exists and results are quickly generated. Although the HBS methodology is not as accurate as 24-h urine excretion or national nutritional surveys, it allows for an approximate estimation and permits monitoring changes in the consumption of sodium in the population [32]. 

## 5. Conclusions

In Costa Rica, in urban and rural zones and across income stratum, the sodium available for consumption at the household level (based on households’ food and beverage acquisitions) considerably exceeds the maximum WHO recommendation. Rural areas and individuals with lower income are those with the greater risk of having high sodium consumption. It is imperative to promote public health interventions in Costa Rica to reduce excessive consumption of sodium in the population, both at home and in the processed foods supply. This must be conducted in order to contribute to the reduction of hypertension and associated chronic diseases and to meet public health interventions [27,28,32,37]. The pattern of sodium consumption by the population of Costa Rica is typical of a developing country. Continued nutritional analysis of the ENIGH database is recommended in order to evaluate and monitor actions to reduce salt/sodium intake in the population. Results need to be confirmed by 24-h urine excretion. In addition, it is desirable that the salt reduction program works in conjunction with the National Salt Fortification Program, as both health policies should strive to work in a synchronized way so that each one achieves its purpose without being to the detriment of the other.

## Figures and Tables

**Table 1:** Household energy and sodium availability according to region and zone of residence in Costa Rica with comparisons of the data from 2004–2005 and 2012–2013.

Zone	*n*		Energy (kcal/Person/Day)		Sodium (g/Person/Day)		Sodium (g/Person/Day/2000 kcal)	
	2004–2005	2012–2013	2004–2005	2012–2013	2004–2005	2012–2013	2004–2005	2012–2013
Costa Rica	1,134,433	1,396,747	2315	2390	3.9	4.6 ^a^	3.4	3.8 ^a^
Urban	705,111	1,023,061	2263	2344	3.6	4.4 ^a,b^	3.2	3.8 ^a,b^
Rural	429,322	373,686	2400	2531	4.5	5.2 ^a,b^	3.8	4.1 ^a,b^

^a^ Statistically significant differences between 2004–2005 and 2012–2013 (*p* < 0.0001); and ^b^ rural vs. urban (*p* < 0.0001).

**Table 2:** Household energy and sodium availability based on food purchases according to increasing quintiles of income distribution in Costa Rica with comparisons of the data from 2004–2005 and 2012–2013.

Quintile	*n*		Energy (kcal/Person/Day)		Sodium (g/Person/Day)		Sodium (g/Person/Day/2000kcal)	
	2004–2005	2012–2013	2004–2005	2012–2013	2004–2005	2012–2013	2004–2005	2012–2013
Costa Rica	1,134,433	1,396,747	2315	2390	3.9	4,6 ^a^	3.4	3.9 ^a^
I	225,773	279,044	1896	1724	3.9	3,8 ^b^	4.1	4.4 ^b^
II	226,647	279,642	2065	2195	3.5	4.7 ^a^	3.4	4.3 ^a^
III	228,332	279,437	2276	2494	4.0	4.7	3.5	3.8
IV	226,503	279,409	2626	2679	4.4	4.9 ^a^	3.4	3.7 ^a^
V	227,178	279,215	2712	2669	3.9	4.7 ^a^	2.9	3.5 ^a^

^a^ Statistically significant differences between 2004–2005 and 2012–2013 (*p* < 0.0001); and ^b^ between quintiles of income (*p* < 0.05).

**Table 3:** Dietary sources of sodium acquired for household consumption (g/person/day) according to increasing quintiles of income distribution based on food purchases in Costa Rica with the comparison of the data from 2004–2005 and 2012–2013.

**2012–2013**								
**Food Group**	***n***	**Costa Rica**		**Quintiles of Per Capita Income Distribution**				
		**g/Person/Day**	**%**	**I**	**II**	**III**	**IV**	**V**
Common salt (table or kitchen)	1,396,747	2.78	60.2	72.4 ^b^	66.3 ^b^	61.6 ^b^	58.2 ^b^	45.2 ^b^
Processed foods	1,396,747	0.65 ^a^	14.2 ^a^	10.1 ^b^	10.9 ^b^	14.1 ^b^	15.8 ^b^	19.4 ^b^
Sodium-based condiments	1,396,747	0.61 ^a^	13.2 ^a^	11.8 ^b^	15.0 ^b^	13.1 ^b^	11.8 ^b^	14.0 ^b^
Ready to eat meals	1,396,747	0.33 ^a^	7.2 ^a^	2.4 ^b^	3.8 ^b^	6.1 ^b^	8.7 ^b^	14.3 ^b^
In natura foods	1,396,747	0.24	5.1	3.4 ^b^	4.0 ^b^	5.2 ^b^	5.5 ^b^	7.3 ^b^
**Total**	1,396,747	4.61	100	100	100	100	100	100
**2004–2005**								
**Food Group**	***n***	**Costa Rica**		**Quintiles of Per Capita Income Distribution**				
		**g/person/day**	**%**	**I**	**II**	**III**	**IV**	**V**
Common salt (table or kitchen)	1,134,433	2.37	60.2	77.2	66.3	62.4	54.7	41.2
Processed foods	1,134,433	0.61	15.4	8.1	13.9	15.3	17.1	22.5
Sodium-based condiments	1,134,433	0.36	9.3	6.4	7.7	9.3	11.7	10.8
Ready to eat meals	1,134,433	0.38	9.8	5.1	7.6	8.0	10.7	17.2
In natura foods	1,134,433	0.21	5.4	3.2	4.6	4.9	5.8	8.3
**Total**	1,134,433	3.94	100	100	100	100	100	100

^a^ Statistically significant differences between food groups in 2012–2013 and 2004–2005 (*p* < 0.05); and ^b^ between food groups in 2012–2013 and 2004–2005 of quintiles of income (*p* < 0.05).
